# Infective endocarditis caused by *Enterobacteriaceae*: phenotypic and molecular characterization of *Escherichia coli* and *Klebsiella pneumoniae* in Rio de Janeiro, Brazil

**DOI:** 10.1007/s42770-021-00528-w

**Published:** 2021-09-22

**Authors:** Nathália L. Andrade, Ana Carolina da Cruz Campos, Andrea Maria Cabral, Paula Hesselberg Damasco, Jerome Lo-Ten-Foe, Ana Cláudia P. Rosa, Paulo V. Damasco

**Affiliations:** 1 Department of Microbiology, Immunology and Parasitology, Biomedical Center, Rio de Janeiro State University, Blv 28 de Setembro, 87, 3th floor, Vila Isabel Rio de Janeiro, Brazil; 2 Department of Medical Microbiology and Infection Prevention, University of Groningen, University Medical Center Groningen, EB80 Hanzeplein 1, 9713, GZ Groningen, The Netherlands; 3 Pedro Ernesto University Hospital, Rio de Janeiro State University, Vila Isabel Rio de Janeiro, Brazil; 4 Antônio Pedro University Hospital, Federal Fluminense University, Niterói, Brazil; 5 Department of Infectious and Parasitic Diseases, Graffrée e Guinle University Hospital, Federal University of the State of Rio de Janeiro, Rio de Janeiro, Brazil

**Keywords:** Endocarditis, Enterobacteriaceae, Biofilm, Adhesion, Next-generation sequencing, Brazil

## Abstract

The etiological agent for infective endocarditis (IE), a life-threatening disease, is usually gram-positive bacteria. However, gram-negative bacteria can rarely cause IE and 4% of cases are associated with morbidity and mortality. This study aimed to characterize *Escherichia coli* and *Klebsiella pneumoniae* isolates from the blood of patients with IE. The characteristics of blood isolates were compared with those of urinary isolates from patients with urinary tract infections (UTIs). The results of this study revealed that *K. pneumoniae* isolates from patients with IE were phylogenetically related to those from patients with UTI. Additionally, the resistance phenotype, resistance gene, virulence gene, and plasmid profiles were similar between the blood and urinary isolates. The isolates belonging to the sequence types (STs) 76, 36, 101 (*K. pneumoniae*), and 69 (*E. coli*) are reported to be associated with drug resistance. The *Enterobacteriaceae* isolates from patients with IE did not produce extended-spectrum β-lactamase or carbapenemase. Additionally, this study investigated the virulence phenotype, biofilm formation ability, and the ability to adhere to the epithelial cells in vitro of the isolates. The isolates from patients with IE exhibited weaker biofilm formation ability than the urinary isolates. All isolates from patients with IE could adhere to the renal epithelial cells. However, three isolates from patients with UTIs could not adhere to the epithelial cells. The closely related *K. pneumoniae* isolates (648, KP1, KP2, KP3, and KP4) could not form biofilms or adhere to the epithelial cells. In summary, the molecular analysis revealed that the genetic characteristics of IE-causing *K. pneumoniae* and *E. coli* were similar to those of UTI-causing isolates. These isolates belonged to the STs that are considered treatable. Genetically similar isolates did not exhibit the same virulence phenotype. Thus, these non-hypervirulent clones must be monitored as they can cause complex infections in susceptible hosts.

**Supplementary Information:**

The online version contains supplementary material available at 10.1007/s42770-021-00528-w.

## Introduction

Infective endocarditis (IE), a systemic life-threatening infection, requires a multidisciplinary therapeutic strategy [1, 2]. The incidence of IE, which is a rare pathological condition, has increased in developed countries but there are limited studies on IE in developing countries [2, 3]. Endocarditis is the fourth most common life-threatening infectious syndrome after urosepsis, pneumonia, and intra-abdominal sepsis in the medical facilities of developed countries [3].

Gram-negative bacteria that cause IE are traditionally classified into the following two main categories: HACEK group (*Haemophilus* spp., *Aggregatibacter* spp., *Cardiobacterium hominis*, *Eikenella corrodens*, and *Kingella kingae*) and non-HACEK group (mostly *Pseudomonas* spp. and members of the *Enterobacteriaceae* family) [4, 5]. *Escherichia coli* and *Klebsiella pneumoniae* are the etiological agents for community-associated and nosocomial infections, especially bloodstream infections and urinary tract infections (UTIs) [6–8]. Recently, there is an increased incidence of invasive infections caused by *E. coli* and *K. pneumoniae* [7–9]. The treatment of *E. coli* and *K. pneumoniae* infections is challenging owing to the development of multidrug resistance in the pathogens [10, 11]. In particular, drug resistance is a major challenge to treat virulent gram-negative bacterial infections [5, 7]. Several studies have analyzed the pathophysiology of the virulence and the mechanisms underlying host cell adhesion of gram-positive microorganisms in IE. However, the pathological mechanisms of IE caused by the members of the family *Enterobacteriaceae* have not been completely elucidated [2–5].

This study aimed to comparatively analyze the molecular characteristics of *E. coli* and *K. pneumoniae* isolated from the blood of patients with IE in Rio de Janeiro, Brazil, with those of other pathogens isolated from the urine of hospitalized patients with UTIs. Additionally, the virulence mechanisms of adherence to host cells were investigated using two different cell lines. Furthermore, the biofilm formation ability of the blood and urinary isolates was examined. The findings of this study will provide novel insights into IE caused by *E. coli* and *K. pneumoniae*, especially multidrug-resistant (MDR) pathogens.

## Material and methods

### Bacterial strains

In this study, five isolates from patients with IE were investigated. The *E. coli* isolates were obtained from the blood samples of an elderly female patient with community-associated IE (CAIE) at the Pedro Ernesto University Hospital (HUPE/UERJ). The *K. pneumoniae* isolates (KP1, KP2, KP3, and KP4) were obtained from the blood samples of an elderly male patient with renal failure and classical healthcare-associated endocarditis (HAIE) at the Hospital of the Public Network, Ordem Terceira do Carmo Hospital, Rio de Janeiro, Brazil. Other pathogenic *K. pneumoniae* strains (1076, 648, 2801, and 5459) were obtained from the urine of hospitalized patients with UTI at the HUPE/UERJ. The EAEC042 and UPECI64 strains were used as positive controls for biofilm formation and adherence assays, respectively. The DH5α strain was used as the negative control in these assays [12]. The sequences of the *E. coli* strains UMN026 (National Center for Biotechnological Information (NCBI) reference sequence: NC_011751.1), JJ1886 (NCBI reference sequence: CP006784.1), UTI89 (NCBI reference sequence: NC_007946.1), 536 (NCBI reference sequence: NC_008253.1), and MG1655 (NCBI reference sequence: NZ_CP032667.1) were downloaded from the NCBI database and used as a control for molecular analysis (Table 1).

**Table 1:** Bacterial isolates, resistance phenotype, resistance, and virulence genotype and adhesion results

Isolates	Species	Source	Hospital	Resistance phenotype	Resistance genotype	Virulence genotype	Adherence Vero	Adherence HEp2
DO7785	*E. coli*	Blood	HUPE	Ampicillin, trimethoprim, trimethoprim sulfamethoxazole	*strA*, *strB*, *aadA5*, *bla*_*TEM-1B*_, *sul1*, *sul2*, and *dfrA17*	*Iuat*, *alls*, *fimH*, *OmpA*, *irp2*	Adhere	Adhere
KP1	*K. pneumoniae*	Blood	OTC	Ampicillin, nitrofurantoin, and fosfomycin	*bla*_*SHV-1*_, *oqxB*, *oqxA*, and *fosA*	*mrkD*, *ureA*, *uge*, *pgaC*, *wzi*, *fimC*, *OmpA*, *wabG*, *hgpA*	Adhere	Adhere
KP2	*K. pneumoniae*	Blood	OTC	Ampicillin, nitrofurantoin, and fosfomycin	*bla*_*SHV-1*_, *oqxB*, *oqxA*, and *fosA*	*mrkD*, *ureA*, *uge*, *pgaC*, *wzi*, *fimC*, *OmpA*, *wabG*, *hgpA*	Adhere	Adhere
KP3	*K. pneumoniae*	Blood	OTC	Ampicillin, nitrofurantoin, and fosfomycin	*bla*_*SHV-1*_, *oqxB*, *oqxA*, and *fosA*	*mrkD*, *ureA*, *uge*, *pgaC*, *wzi*, *fimC*, *OmpA*, *wabG*, *hgpA*	Adhere	Adhere
KP4	*K. pneumoniae*	Blood	OTC	Ampicillin, nitrofurantoin, and fosfomycin	*bla*_*SHV-1*_, *oqxB*, *oqxA*, and *fosA*	*mrkD*, *ureA*, *uge*, *pgaC*, *wzi*, *fimC*, *OmpA*, *wabG*, *hgpA*	Adhere	Adhere
2801	*K. pneumoniae*	Urine	HUPE	Cefuroxime, Cefotaxime, ceftazidime, gentamicin, tobramycin, ciprofloxacin, nitrofurantoin, fosfomycin, and trimethoprim	*bla*_*TEM-1B*_, *bla*_*CTXM-15*_, *bla*_*OXA-1*_, *oqxA*, *oqxB*, *fosA*, *aac(3)lld*, *aac(6′)lb-cr*, *catB4*, *tet(D)*, *dfrA14*, and *acrR*	*ybtS*, *mrkD*, *ureA*, *uge*, *pgaC*, *wzi*, *OmpA*, *wabG*, *hgpA*, *irp2*	Adhere	Adhere
5459	*K. pneumoniae*	Urine	HUPE	Ampicillin and nitrofurantoin	*bla*_*OXA-10*_, *oqxB*, *oqxA*, and *fosA*	*mrkD*, *ureA*, *uge*, *pgaC*, *wzi*, *fimC*, *OmpA*, *wabG*, *hgpA*	Adhere	Adhere
648	*K. pneumoniae*	Urine	DASA	Cefuroxime, cefotaxime, ceftazidime, gentamicin, tobramycin, ciprofloxacin, nitrofurantoin, fosfomycin, and trimethoprim	*bla*_*SHV-1*_, *bla*_*CTXM-164*_, *oqxB*, *oqxA*, *fosA*, and *acrR*	*mrkD*, *ureA*, *uge*, *pgaC*, *wzi*, *fimC*, *OmpA*, *wabG*, *hgpA*	Non-adhere	Non-adhere
1076	*K. pneumoniae*	Urine	HUPE	Amoxicillin	*bla*_*SHV-11*_, *oqxB*, *oqxA*, and *fosA5*	*mrkD*, *ureA*, *uge*, *pgaC*, *wzi*, *fimC*, *OmpA*, *wabG*, *hgpA*	Adhere	Adhere

*HUPE*, Pedro Ernesto University Hospital; *OTC*, Ordem Primeira do Carmo Hospital; *DASA*, Diagnóstico da América

### Strain isolation, identification, culture conditions, and susceptibility test

The strains were plated onto the following selective media: eosin methylene-blue agar (Bevton & Dickson, NJ, USA) and MacConkey agar (Becton & Dickson, NJ, USA). Biochemical and matrix-assisted laser desorption/ionization time-of-flight mass spectrometry (Bruker, Germany) analyses were performed to identify the strains. The strains were stored in GC medium with 20% glycerol at − 20 °C and − 70 °C. The isolates were cultured in blood agar medium for 18 h at 37 °C. The drug susceptibility assays were performed using VITEK-2 (bioMérieux, Marcy l’Etoile, France), following the European Committee on Antimicrobial Susceptibility Testing guidelines and the results were confirmed using the E-test (bioMérieux) assays. The antimicrobial resistance classification of isolates was defined according to the standardized international terminology established by the European Centre for Disease Prevention and Control and the Centers for Disease Control and Prevention as follows: MDR, non-susceptibility to at least one agent in three or more antimicrobial categories; extensively drug-resistant (XDR), non-susceptibility to at least one agent in all but two or fewer antimicrobial categories; pan-drug-resistant, non-susceptibility to all agents in all antimicrobial categories [13].

### DNA extraction, whole-genome sequencing, assembly, and annotation

Total bacterial DNA was extracted from each isolate using the Ultraclean® microbial DNA isolation kit (MO BIO Laboratories, Carlsbad, CA, USA), following the manufacturer’s instructions. The DNA library was prepared using the Illumina Nextera XT kit. The sequences were assembled using CLC Genomics Workbench v10.0.1 (CLC bio A/S, Aarhus, Denmark) with default settings and optimal word size. The assembled sequences were annotated using RAST server version 2.0 [14] (see Supplementary Data S1).

### Sequencing data analysis

The assembled genomes were uploaded in the FASTA format to the Center for Genomic Epidemiology (CGE) multi-locus sequence typing (MLST) finder website (version 1.7) to identify the sequence types (STs) of the isolates [15]. The presence of antibiotic-resistant genes was determined by uploading the assembled genomes in FASTA format to ResFinder 2.1 [16] from the CGE server. The virulence genes were identified using the Basic Local Alignment Search Tool of the NCBI or European Nucleotide Archive database with the CLC Genomics Workbench v10.0.1 (CLC bio A/S, Aarhus, Denmark) tool (see the complete list of virulence genes in Supplementary Data S1 and S2). The serotypes of *K. pneumoniae* and *E. coli* isolates were predicted using the Kaptive web tool [17] and SerotypeFinder tool, respectively [18]. To determine the phylogenetic characteristics, the sequences were uploaded into SeqSphere v.4.1.9 (Ridom, Munster, Germany) and genotyped using a gene-by-gene typing approach with a 2358-gene core-genome MLST (cgMLST) scheme. The plasmid replicon types were identified by uploading the genome sequences of the isolate to the PlasmidFinder v.2.0.1 [19] webtool. The plasmids in the isolates were reconstructed using the MOB-suite tool [20].

### Cell lines and cell culture conditions

The Vero cell line (American Type Culture Collection (ATCC) CCL-81; derived from African green monkey kidney) was used to mimic the normal human renal epithelial cells. The HEp-2 cell line (ATCC CCL 23; derived from human laryngeal epithelial cells) was used as a positive control for the adherence assay [21]. The Vero and HEp-2 cells were cultured in minimum essential medium (MEM, Gibco-BRL) supplemented with 5% v/v fetal calf serum (Gibco-BRL), D-mannose, 50 µg/mL gentamicin, and 2.5 µg/mL amphotericin B. The cells were cultured on 13-mm diameter glass coverslips placed in 24-well tissue culture plates (Costar) to obtain the sub-confluent cell monolayer. Next, the monolayer was washed twice with Dulbecco’s phosphate-buffered saline (PBS-D; pH 7.2) and incubated with 1 mL of fresh MEM without antibiotics.

### Adherence assays

The cell monolayers were incubated with 35 µL of bacterial culture cultured overnight in Luria–Bertani (LB) medium for 3 or 6 h at 37 °C and 5% CO_2_*.* In the 6-h assay, the cells were washed with PBS-D and fresh medium was added after 3 h. The cells were washed twice with PBS-D to remove the non-adherent bacteria, fixed with methanol, and stained with 5% Giemsa stain for 30 min. The coverslips were removed from the wells and the cells were washed with water, dried, mounted on glass slides, and examined using oil immersion microscopy [22].

### Quantitative biofilm formation assay

The ability of *E. coli* and *K. pneumoniae* strains to form biofilms on polystyrene surfaces was examined according to the methodology of Sheikh et al. (2001) [12] with modifications. The bacterial suspension (5 µL) cultured overnight in LB medium at 37 °C with shaking was incubated with 200 µL of Dulbecco’s minimal essential medium (D-MEM, Gibco-BRL) in 96-well microtiter plates at 37 °C overnight. The culture was rinsed twice with 200 µL PBS to remove the planktonic cells. The biofilm was stained with crystal violet and the stain was solubilized with 200 µL of 95% ethanol for 2 min at 37 °C room temperature. Next, 150 µL of the mixture was transferred to a new microtiter plate and the absorbance at 570 nm was measured using an enzyme-linked immunosorbent assay plate reader. The assay was performed in triplicate and three independent experiments were performed [23]. The average optical density (OD) was calculated for all strains, including the negative control (DH5α strain), according to the criteria reported by Stepanovic et al. [23]. The cut-off value was three standard deviations (SDs) from the mean OD of the negative control (ODc average = OD of negative control (3 × SD of negative control)). To interpret the results, the strains were divided into the following categories: no biofilm producer (0), weak biofilm producer (+ or 1), moderate biofilm producer (+ + or 2), and strong biofilm producer (+ +  + or 3). This classification was based on the following criteria: OD ≤ ODc, no biofilm producer; ODc < OD ≤ 2 × ODc, weak biofilm producer; 2 × ODc < OD ≤ 4 × ODc, moderate biofilm producer; 4 × ODc < OD, strong biofilm producer. All data were analyzed using GraphPad Prism. The data were analyzed using one-way analysis of variance. The differences were considered significant at *p* < 0.0001.

### Nucleotide sequence accession number

The sequencing data of isolates were deposited in the NCBI public database (project number: PRJNA657729) (see Supplementary Data S3 for individual accession numbers).

## Results

### Resistance phenotype

The origin and species of the strains used in this study are listed in Table 1. The *E. coli* isolate DO7785 was resistant to trimethoprim, trimethoprim/sulfamethoxazole, and ampicillin. Six isolates were classified as MDR in this study (the exceptions were the isolates 5459 and 1076). KP1, KP2, KP3, and KP4 were resistant to ampicillin, nitrofurantoin, and fosfomycin. The isolate 1076 was resistant to amoxicillin, whereas the isolates 2801 and 648 were resistant to piperacillin/tazobactam, cefuroxime, cefotaxime, ceftazidime, gentamicin, tobramycin, ciprofloxacin, nitrofurantoin, fosfomycin, trimethoprim, and ciprofloxacin. The isolate 5459 was resistant to ampicillin and nitrofurantoin (Table 1).

### Detection of antibiotic-resistant genes

The *E. coli* isolate DO7785 harbored the following resistance genes: *strA*, *aadA5*, *strB*, *bla*_*TEM-1B*_, *sul1*, *sul2*, and *dfrA17*. The KP1, KP2, KP3, and KP4 isolates harbored the following resistance genes: *bla*_*SHV-1*_, *oqxB*, *oqxA*, and *fosA*. The isolate 1076 harbored the following resistance genes: *bla*_*TEM-1B*_, *oqxB*, *oqxA*, and *fosA5*. The isolate 648 harbored the following resistance genes: *bla*_*SHV-1*_, *bla*_*SHV-164*_, *oqxB*, *oqxA*, *fosA*, and *acrR*. The isolate 5459 harbored the following resistance genes: *bla*_*OXA-10*_, *oqxB*, *oqxA*, and *fosA*. The isolate 2801 harbored the following resistance genes: *bla*_*TEM-1B*_, *bla*_*CTXM-15*_, *bla*_*OXA-1*_, *oqxB*, *oqxA*, *fosA*, *aac(3)lld*, *aac(6′)lb-cr*, *catB4*, *tet(D)*, *dfrA14*, and *acrR*. Although most isolates harbored the resistance genes for fosfomycin, only three were susceptible to fosfomycin (Table 1).

### Molecular typing and phylogenetic analysis

**Fig. 1:**
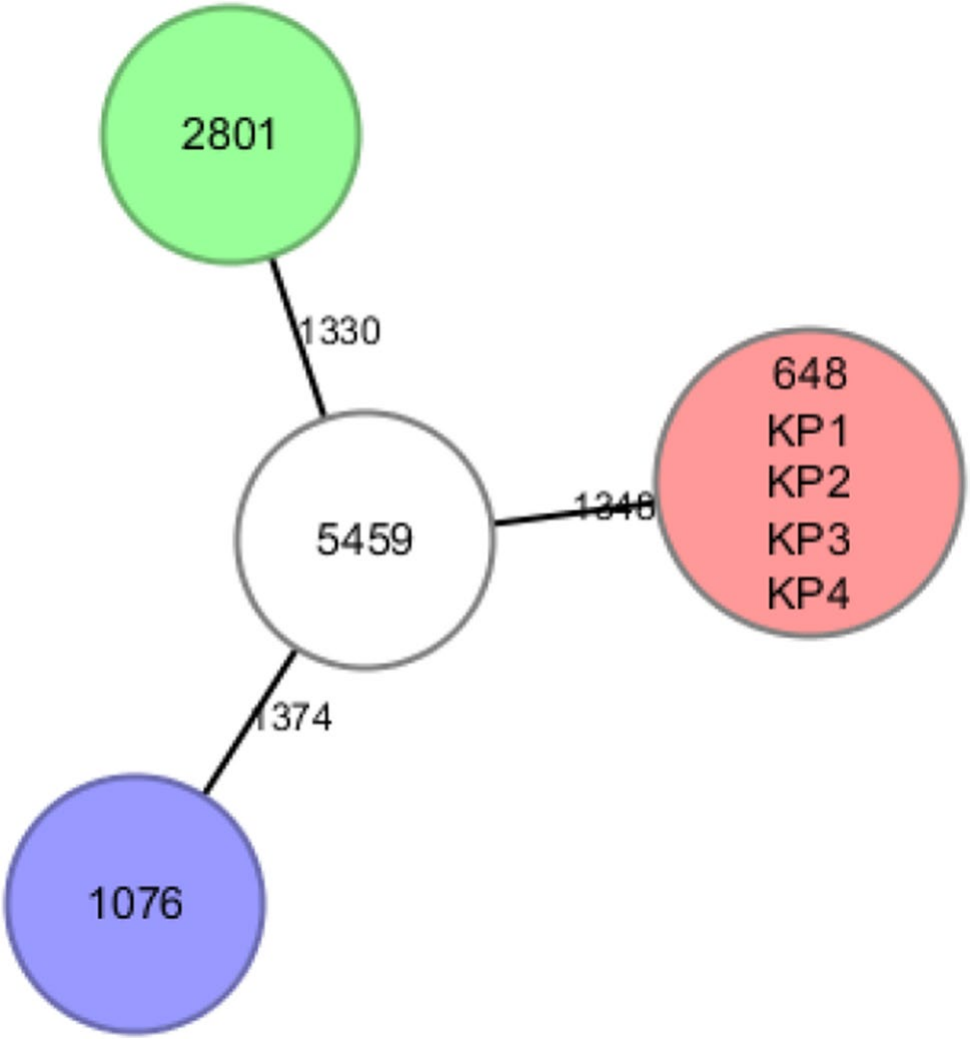
Neighbor-joining (NJ) phylogenetic trees of isolates. The results using cgMLST based on 2358 genes showed the KP isolates (from endocarditis) and the 648 isolate (from urine), in red, belongs to the ST76 type. In green, the isolate 2801 (from urine) belongs to the ST101. In purple, the isolate 1076 (from urine) belongs to ST36. In white is 5459, another *K. pneumoniae* isolate from urine for which it was not possible to identify the ST type. The figure also showed the distance in genes between the *K. pneumoniae* isolates

All isolates were subjected to whole-genome sequencing to analyze the phylogenetic relationship and molecular characteristics. The *E. coli* DO7785 isolate belonged to ST69 and serotype O153:H2, whereas all *K. pneumoniae* isolates belonged to ST76. The urinary *K. pneumoniae* isolates belonged to ST36 and ST101. The *K. pneumoniae* isolates from patients with IE and the isolate 648 from patients with UTI belonged to ST76. All *K. pneumoniae* isolates were classified into the serotype K2. The urinary isolate 648 and the blood *K. pneumoniae* isolates (from patients with IE) were closely related although they infected different sites. The cgMLST scheme revealed that the clustering of the isolate 648 and the *K. pneumoniae* isolates from patients with IE was distinct from that of other urinary *K. pneumoniae* isolates. Similarly, *E. coli* DO7785 clustered with the urinary reference strain UMN26, which indicated a high degree of genetic relatedness between these two strains (Fig. 1).

### Plasmid and virulence gene analyses

The presence of plasmids in *E. coli* and *K. pneumoniae* isolates was examined. In the *E. coli* isolate DO7785, a large plasmid sequence (95,458 bp) containing two replicon types (IncFIB and IncFII) was identified. All *K. pneumoniae* isolates and the isolate 648 harbored the same reconstructed plasmid containing the replicon type Incl1, whereas the isolate 2801 harbored a different plasmid that contained both IncFIB and IncFII replicons. The other two *K. pneumoniae* isolates (5459 and 1076) did not harbor any plasmid. Interestingly, the plasmids in *E. coli* DO7785 and *K. pneumoniae* 2801 did not harbor any resistance genes. However, the IncFIA plasmids in the *K. pneumoniae* isolates and the closely related isolate 648 contained beta-lactam antibiotic resistance genes. Additionally, virulence genes were identified in the plasmids, including genes involved in copper resistance and iron uptake (see Supplementary Data S4 for complete plasmid sequences). Eight of the 17 virulence genes identified were present in the study isolates. All isolates contained *mrkD*, *urea*, *uge*, *pgaC*, *fimC*, *ompA*, and *hgpA*. In contrast, *ybtS* and *irp2* were detected only in the isolate 2801. The urinary and blood isolates exhibited identical virulence gene profiles (see Supplementary Material S5).

### Quantification of biofilm formation

**Fig. 2:**
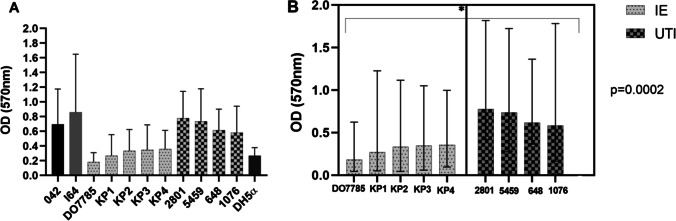
Quantitative biofilm results. (A) The strains indicated in light grey with dots are isolates from the blood whereas the strains indicated in dark grey and squares are from urine samples. The strains 042 (EAEC) and I64 (UPEC) are positive controls, whereas the strain DH5α is a negative control. The strains were divided into categories based upon the previously calculated OD values OD ≤ ODc = no biofilm producer; ODc < OD ≤ 2xODc = weak biofilm producer; 2xODc < OD ≤ 4xODc = moderate biofilm producer; 4xODc < OD = strong biofilm producer, being the ODc the OD values for the negative control strain. (B) Comparison between the isolates from blood (IE) and the isolates from urine (UTI). The asterisk indicates a statistically significant difference between all the isolates from blood and urine (p < 0.05)

The biofilm-forming ability of the clinical isolates was examined. The *E. coli* DO7785 isolate could not form biofilms. KP1, KP2, KP3, and KP4 exhibited a weak biofilm-forming ability. In contrast, the genetically closely related isolate 2801 exhibited a moderate biofilm-forming ability (Fig. 2A). Compared with the blood isolates, the urinary isolates formed significantly stronger biofilms (*p* < 0.0001). Among the urinary isolates, the isolate 5459 exhibited a strong biofilm-forming ability, whereas the isolates 648 and 1076 exhibited a moderate biofilm-forming ability. These results indicated that the urinary isolates exhibited a higher biofilm-forming ability than the blood isolates (Fig. 2B).

### Adherence of Escherichia coli and Klebsiella pneumoniae strains to Vero cell and HEp-2 cell lineages

Adherence assays were performed using the urothelial and laryngeal epithelial cells with the positive control strains EAEC042 and UPECI64. The *E. coli* isolate DO7785 adhered to the Vero and HEp-2 cell lines. All *K. pneumoniae* blood isolates (KP1, KP2, KP3, and KP4) adhered to the Vero and HEp-2 cell lines. Additionally, the adherence ability of *K. pneumoniae* urinary isolates to these cell lines was examined. The isolates 2801 and 1076 adhered to the epithelial cells, while the isolate 5459 poorly adhered to the epithelial cells. The isolate 648 did not adhere to the Vero or HEp-2 cells (See Supplementary Data S6 for the data on pathogen adherence to Vero and HEp-2 cells) (Table 1).

## Discussion

The incidence of IE caused by the members of the family *Enterobacteriaceae* in community and hospital settings is rare. However, a previous prospective study reported that the incidence of IE caused by gram-negative bacteria in Rio de Janeiro, Brazil, was 8.2% in the 8-year study period [3]. Another study reported that the incidence of IE caused by gram-negative bacteria in two principal teaching hospitals in Rio de Janeiro, Brazil, was higher than that reported in other studies [2]. A prospective study in Italy reported that *E. coli* was the most common etiological agent of IE caused by non-HACEK gram-negative bacteria, followed by *Pseudomonas aeruginosa* and *K. pneumoniae* [5]. Here, the characteristics of *E. coli* isolate (DO7785) from patients with CAIE and *K. pneumoniae* isolates from patients with HAIE were compared with those of urinary isolates to understand the pathophysiology of endocarditis caused by *Enterobacteriaceae*.

The results of this study demonstrated that the *E. coli* isolate DO7785 was resistant to the four tested antibiotics. However, the *E. coli* isolate DO7785 did not produce extended-spectrum β-lactamase (ESBL) and was not resistant to fosfomycin. The four *K. pneumoniae* clinical isolates were resistant to only three tested antibiotics and did not produce ESBL. In contrast to *E. coli* DO7785, the four *K. pneumoniae* isolates were resistant to fosfomycin. These results were consistent with those of previous studies, which reported that ESBL-producing or carbapenemase-producing *Enterobacteriaceae* members rarely cause IE [24]. *E. coli* DO7785 exhibited resistance to ampicillin and trimethoprim/sulfamethoxazole. A previous study reported a high prevalence of ampicillin resistance in *E. coli* [25]*.* Intravenous administration of fosfomycin has been suggested as a potential treatment for infections caused by drug-resistant gram-negative bacteria, including endocarditis. Fosfomycin has been considered as an alternative antimicrobial to treat infections caused by MDR gram-negative bacteria in cases where other antibiotic treatment options have failed [26]. Most isolates in this study were classified as MDR. This was not consistent with the findings of previous studies, which reported that endocarditis caused by *Enterobacteriaceae* can be successfully treated using first-choice antibiotics [27, 28]. In contrast, another study reported a case of endocarditis caused by carbapenemase-resistant *K*. *pneumoniae*, which has raised concerns about endocarditis caused by MDR bacteria [29]*.*

Additionally, the molecular characteristics of isolates from patients with IE and urinary isolates were comparatively analyzed. The four *K. pneumoniae* isolates (KP1, KP2, KP3, and KP4) belonged to ST76, whereas *E. coli* DO7785 belonged to ST69, which is commonly associated with UTI cases. The *K. pneumoniae* urinary isolate 648 from a patient with UTI also belonged to ST76. The cgMLST scheme revealed that the isolate 648 and the four KP isolates exhibited similar clustering patterns. Moreover, ST76 *K. pneumoniae* isolates have been previously reported to be associated with infection outbreaks in hospitals [30, 31]. These ST76 isolates exhibited the same plasmid, resistance, and virulence gene profiles. This indicated that the complex UTI is a potential source of endocarditis-causing *K. pneumoniae* in the patient. The other *K. pneumoniae* isolates belonged to ST36 and ST101, which are globally associated with drug-resistant and hypervirulent *K. pneumoniae* isolates from hospitalized patients with UTIs. ST101 and ST36 infections were reported to be the etiological agents for invasive infections in humans [32]. The molecular characteristics of *E. coli* DO7785 were compared with those of the reference urinary isolate obtained from a public database. The results of this study revealed similarities between the blood *E. coli* isolate from a patient with IE and a urinary isolate belonging to the same ST. In contrast to the findings of previous studies, this study reported that the isolates do not harbor *rmpA*, which is detected in hypervirulent *K. pneumoniae* belonging to the same ST. Additionally, the isolates did not exhibit the same drug resistance profile as that of the hypervirulent *K. pneumoniae* [33]. This indicated that the isolates are not hypervirulent and that they do not produce ESBL, which can be a major threat to human health as they can cause invasive infections [34].

The *E. coli* DO7785 isolate harbored genes associated with aminoglycoside resistance (*strA*, *strB*, and *aadA5*), beta-lactam resistance (*bla*_*TEM-1B*_), sulfonamide resistance (*sul1* and *sul2*), and trimethoprim resistan*ce* (*dfrA17*). Araby et al. (2015) [35] demonstrated that sulfonamide resistance genes were prevalent among ESBL-producing *E. coli* and non-ESBL-producing *E. coli* with a high frequency of *bla*_*TEM*_ [35]. *dfrA17*, which was expressed in the isolate *E. coli* DO7785, is commonly found in Korea [36]. All clinical *K. pneumoniae* isolates exhibited similar resistance gene profiles (beta-lactamase resistance (*bla*_*SHV-1*_), fosfomycin resistance, and *oqxA* and *oqxB*). These results are consistent with the resistance phenotype. All *K. pneumoniae* urinary isolates contained the same quinolone resistance genes (*oqxA*, and *oqxB*)*.* The most important mechanism of quinolone resistance is aberrations in the chromosomal regions encoding quinolone resistance [37]. Some studies have suggested that *oqxA* and *oqxB* are conserved in the *K. pneumoniae* chromosomes [38–40]. However, Matinez et al. [41] reported that *oqxA* and *oqxB* are encoded in a large plasmid (< 160 kb) [41]. The presence of *oqxA* and *oqxB* in the plasmids in *K. pneumoniae* can be explained by their capture from the chromosomal genome, which may be a reservoir for this antibiotic resistance [38, 39]. The activity of ESBLs is correlated with fluoroquinolone resistance [42]. The presence of ESBL and some fluoroquinolone-resistant genes in the same mobile genetic elements may result in co-resistance to *β*-lactams and fluoroquinolones [43]. Azargun et al. [42] demonstrated that resistance to fluoroquinolones (89.3%) in ESBL-producing isolates was significantly higher than that in non-ESBL-producing isolates. This indicated that *oqxB* and *oqxA* are detected in a significant proportion of ESBL-producing *Enterobacteriaceae* [42].

Although the urinary *K. pneumoniae* isolate 1076 contained *fosA5* and quinolone resistance genes, it was resistant to only amoxicillin (owing to the presence of *bla*_*SHV11*_, a narrow-spectrum beta-lactamase). In this study, two urinary *K. pneumoniae* isolates (2801 and 648) harbored ESBL-encoding genes belonging to the CTX-M group. The genes located on the plasmids can be easily transmitted [44]. Quinolone resistance among *K. pneumoniae* clinical isolates is a serious health concern as ciprofloxacin is widely prescribed as a broad-spectrum antimicrobial agent for the treatment of UTI caused by ESBL-producing *K. pneumoniae* [45]. Some plasmids co-exist with *bla*_*CTXM*_ and *aac(6′)-Ib-cr*, which promote resistance to cephalosporins and fluoroquinolones [35].

The presence of virulence genes was also investigated in this study because of their importance in the pathogenesis of *E. coli* and *K. pneumoniae* infections. The urease virulence factor, which is critical for catheter encrustation, is encoded in all *K. pneumoniae* from patients with IE and UTIs. Other virulence genes such as *uge*, *fimC*, and *hgpA* identified in these isolates are reported to be associated with the pathogenesis of UTIs [46–48]. Several *Enterobacteriaceae* species form biofilms, which further contribute to the resistance to antimicrobial agents. Biofilms are also associated with device-related infections, including endocarditis and UTIs [49]. In this study, all urinary isolates could form biofilms. In contrast, the blood *E. coli* DO7785 isolate could not form biofilms. Meanwhile, the KP1, KP2, KP3, and KP4 isolates formed weak biofilms. Interestingly, the urinary isolate 2801, which is closely related to the clinical *K. pneumoniae* isolates, formed significantly stronger biofilms than the blood *K. pneumoniae* isolates.

The results of the molecular assay revealed that all strains expressed important virulence genes associated with biofilm formation. *mrkD*, an operon that encodes type 3 fimbriae, plays an important role in biofilm formation [50–53]. The presence of *fimC*, which encodes the chaperone FimC, was also detected. FimC is part of a complex group of adhesion molecules from type 1 fimbriae that is involved in adherence to the host cells and biofilm formation [46]. *E. coli* DO7785 expresses a virulence gene that is commonly present in uropathogenic *Escherichia coli* (UPEC). *fimH* encodes the protein FimH, which is localized at chaperone fimbriae type 1 and promotes the bacterial adherence to the host bladder. Additionally, FimH is involved in biofilm formation and adherence to urinary tissue [54, 55].

Previous epidemiological studies have reported that the low frequency of IE caused by *Enterobacteriaceae* can be attributed to of inability of gram-negative bacteria to attach to the cardiac valves. In contrast, typical gram-positive pathogens and gram-negative HACEK bacterial group can bind to the cardiac valves [5]. In this study, all strains associated with IE adhered to the HEp-2 and Vero cell lines. Among the isolates associated with urinary infections, three *K. pneumoniae* strains exhibited strong adherence, one *K. pneumoniae* strain exhibited poor adherence, and one *K. pneumoniae* could not adhere to the HEp-2 and Vero cell lines. These results suggest that the adherence ability of *Enterobacteriaceae* isolates from patients with IE was different from that reported in previous studies [5]*. E. coli* (DO7785) and all *K. pneumoniae* strains express OmpA, which is an outer membrane porin that is associated with adherence to Vero and HEp-2 cell lines. In addition to encoding the major porin on the outer membrane of gram-negative bacteria, OmpA is involved in the pathogenic mechanisms, such as inhibition of proinflammatory cytokine production in isolated monocytes, induction of cell death, and adherence, invasion, and persistence to host cells [56–58].

## Conclusions

The results of this study revealed that the molecular characteristics of *E. coli* and *K. pneumoniae*, which cause endocarditis, are similar to those of isolates from UTI cases. The virulence and drug resistance profiles of these isolates indicate that they can infect both the urinary system and the cardiovascular system. These isolates belong to STs that are considered a threat to human health. Although the isolates did not exhibit the same resistance and virulence gene profiles as those reported in hypervirulent clones, they can cause invasive infections. The genetically similar isolates did not exhibit the same virulence phenotype. Phenotypic analysis was performed to analyze the pathogenicity of the strains. The isolates formed biofilms and adhered to the host cells, which indicated that they are pathogenic. These results suggest that urine is the source of isolates from patients with IE and that these isolates are not similar to hypervirulent clones. Hence, these isolates must be monitored as they can cause complex infections in susceptible hosts. The limitations of this study include the small sample size. Further studies are necessary to clarify the molecular profile of *E. coli* and *K. pneumoniae* strains from patients with UTIs that can cause IE.

## Supplementary Information

Below is the link to the electronic supplementary material.

Supplementary file1 (PDF 2046 KB)

## Abbreviations

IE: Infective endocarditis
UTIs: Urinary tract infections
CAIE: Community-associated IE
HAIE: Healthcare-associated IE
MDR: Multidrug-resistant
