# Supplementary material for: Infective endocarditis caused by Enterobacteriaceae: phenotypic and molecular characterization of Escherichia coli and Klebsiella pneumoniae in Rio de Janeiro, Brazil
Source: Braz J Microbiol. 2021 Sep 22;52(4):1887–96. doi: 10.1007/s42770-021-00528-w (PMC8578509; doi:10.1007/s42770-021-00528-w)
Supplement: Supplementary file 1 — Supplementary file1 (PDF 2046 KB) [file 42770_2021_528_MOESM1_ESM.pdf]

Infective Endocarditis Caused by Enterobacteriaceae: Phenotypic and Molecular Characterization of *Escherichia coli* and *Klebsiella pneumoniae* Causing Endocarditis in Rio de Janeiro, Brazil

Nathália L. Andrade, Ana Carolina da C. Campos, Andrea Maria Cabral, Paula H. Damasco, Jerome Lo-Ten-Foe, Ana Cláudia P. Rosa, Paulo V. Damasco

Correspondence: Ana Claudia de Paula Rosa Ignácio

Departamento de Microbiologia, Imunologia e Parasitologia, Faculdade de Ciências Médicas, Universidade do Estado do Rio de Janeiro, Brazil. Blv 28 de Setembro, 87 fundos 3 andar, Vila Isabel, Rio de Janeiro.

Postal code: 20551-030 Tel: +55 21 2868-8200| Fax: +55 21 2868-8376

E-mail: anarosa2004@gmail.com

Supplementary data

S.1. List of virulence genes investigated for *E. coli* isolates in this study.

Gene	Gene description[a]
astA	Heat-stable enterotoxin 1
bfpA	Major subunit of bundle-forming pili
cba	Colicin B
ccI	Cloacin
cdtB	Cytolethal distending toxin B
celb	Endonuclease colicin E2
cfa_c	Colonization factor antigen I
cif	Type III secreted effector
cma	Colicin M
cnf1	Cytotoxic necrotizing factor
cofA	Longus type IV pilus subunit
eae	Intimin
eatA	Serine protease autotransporters of *Enterobacteriaceae* (SPATE)
efa1	EHEC factor for adherence
ehxA	Enterohemolysin
epeA	Serine protease autotransporters of *Enterobacteriaceae*
espA	Type III secretion system
espB	Secreted protein B
espC	Serine protease autotransporters of *Enterobacteriaceae*
espF	Type III secretion system
espI	Serine protease autotransporters of *Enterobacteriaceae*
espJ	Prophage-encoded type III secretion system effector
espP	Putative exoprotein precursor
etpD	Type II secretion protein
f17A	Subunit A of F17 fimbrial protein
f17G	Adhesin subunit of F17 fimbriae
fanA	Involved in biogenesis of K99/F5 fimbriae
fasA	Fimbrial 987P/F6 subunit
fedA	Fimbrial protein F107 subunit A
fedF	Fimbrial adhesin AC precursor
fim41a	Mature Fim41a/F41 protein
gad	Glutamate decarboxylase

hlyE	Avian *E. coli* hemolysin
iha	Adherence protein
ipaD	Invasion protein *Shigella flexneri*
ipaH9.8	Invasion plasmid antigen
ireA	Siderophore receptor
iroN	Enterobactin siderophore receptor protein
iss	Increased serum survival
K88ab	K88/F4 protein subunit
katP	Plasmid-encoded catalase peroxidase
lngA	Longus type IV pilus
lpfA	Long polar fimbriae
ltcA	Heat-labile enterotoxin A subunit
mchB	Microcin H47 part of colicin H
mchC	MchC protein
mchF	ABC transporter protein MchF
mcmA	Microcin M part of colicin H
nfaE	Diffuse adherence fibrillar adhesin gene
nleA	Non-LEE-encoded effector A
nleB	Non-LEE-encoded effector B
nleC	Non-LEE-encoded effector C
perA	EPEC adherence factor
pet	Autotransporter enterotoxin
pic	Serine protease autotransporters of *Enterobacteriaceae*
prfB	P-related fimbrial regulatory gene
rpeA	Serine protease autotransporters of *Enterobacteriaceae*
sat	Serine protease autotransporters of *Enterobacteriaceae*
senB	Plasmid-encoded enterotoxin
sepA	Serine protease autotransporters of *Enterobacteriaceae*
sfaS	S-fimbrial minor subunit
sigA	Serine protease autotransporters of *Enterobacteriaceae*
sta1	Heat-stabile enterotoxin ST-Ia
stb	Heat-stabile enterotoxin II
stx1A	Shiga-like toxin 1 A-subunit
stx1B	Shiga-like toxin 1 B-subunit
stx2A	Shiga toxin 2 subunit A
stx2B	Shiga toxin 2 subunit B
subA	Subtilase toxin subunit
saa	STEC autoagglutinating adhesin
tccP	Tir cytoskeleton coupling protein
tir	Translocated intimin receptor protein
toxB	Toxin B
tsh	Serine protease autotransporters of *Enterobacteriaceae*
vat	Serine protease autotransporters of *Enterobacteriaceae*
virF	VirF transcriptional activator
tccP	Tir cytoskeleton coupling protein
tir	Translocated intimin receptor protein
toxB	Toxin B
tsh	Serine protease autotransporters of *Enterobacteriaceae*
vat	Serine protease autotransporters of *Enterobacteriaceae*
virF	VirF transcriptional activator

[a]LEE, locus of enterocyte effacement. The threshold for all the genes in this table was 90% identity and 60% length.

S.2. List of virulence genes investigated for *K. pneumoniae* isolates in this study.

Gene	Gene description
ybtS	Siderophore ybtS
mrkD	MrkD (cell adhesion)
rmpA	RmpA (regulation of transcription)
iutA	Ferric aerobactin receptor IutA
allS	HTH-type transcriptional activator Alls (DNA binding transcription)
fur	Ferric uptake regulation protein
fimH	Type 1 fimbrin D-mannose specific adhesion
ureA	Urease subunit
uge	Uridine diphosphate 4-epimerase
pgaC	Poly-beta-1.6-N-acetyl-D-glucosamine synthase
wzi	Surface assembly of capsule
fimC	Chaperone protein FimC
ompA	Outer membrane protein A
iroN	Ferric enterobactin receptor
wabG	WabG protein
hgpA	Hemin receptor
irp2	Iron regulatory protein

The threshold for all the genes in this table was 90% identity and 60% length.

S.3. Genomic data from *E. coli* and *K. pneumoniae* isolates, %CG, number of contigs, Genome size, N50, and accession number.

**Isolates**	**Specie**	**%CG**	**Number of contigs**	**Genome Size (kb)**	**N50**	**Accession number**
**DO7785**	*Escherichia coli*	50.75	113	5.271	75201	SAMN15341634
**KP1**	*Klebsiella pneumoniae*	56.38	92	5.586	55506	SAMN15341630
**KP2**	*Klebsiella pneumoniae*	56.60	78	5.582	88252	SAMN15341631
**KP3**	*Klebsiella pneumoniae*	53.07	75	5.585	24462	SAMN15341632
**KP4**	*Klebsiella pneumoniae*	56.62	91	5.585	66183	SAMN15341633
**2801**	*Klebsiella pneumoniae*	55.91	350	5.677	34413	SAMN15341628
**5459**	*Klebsiella pneumoniae*	57.95	193	5.061	148506	SAMN15341629
**648**	*Klebsiella pneumoniae*	57.04	248	5.576	142592	SAMN15341627
**1076**	*Klebsiella pneumoniae*	57.37	818	5.278	58459	SAMN15341626

S.4. Plasmids genetic structures identified among isolates in this study.

S.4.1. Plasmid present in the 648, KP1, KP2, KP3 and KP4 *K. pneumoniae* isolate

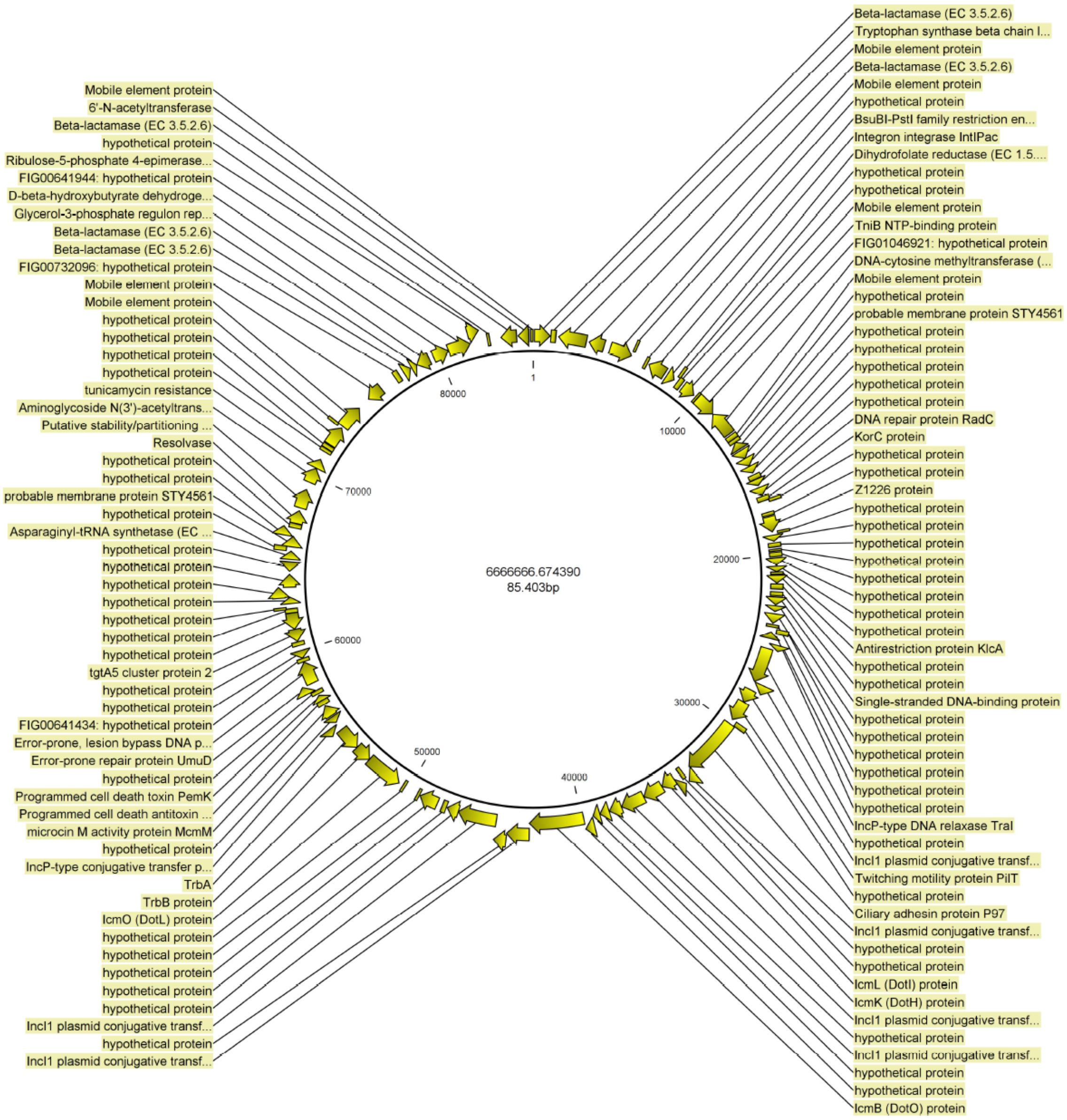

S.4.2. Plasmid present in the 2801 *K. pneumoniae* isolate

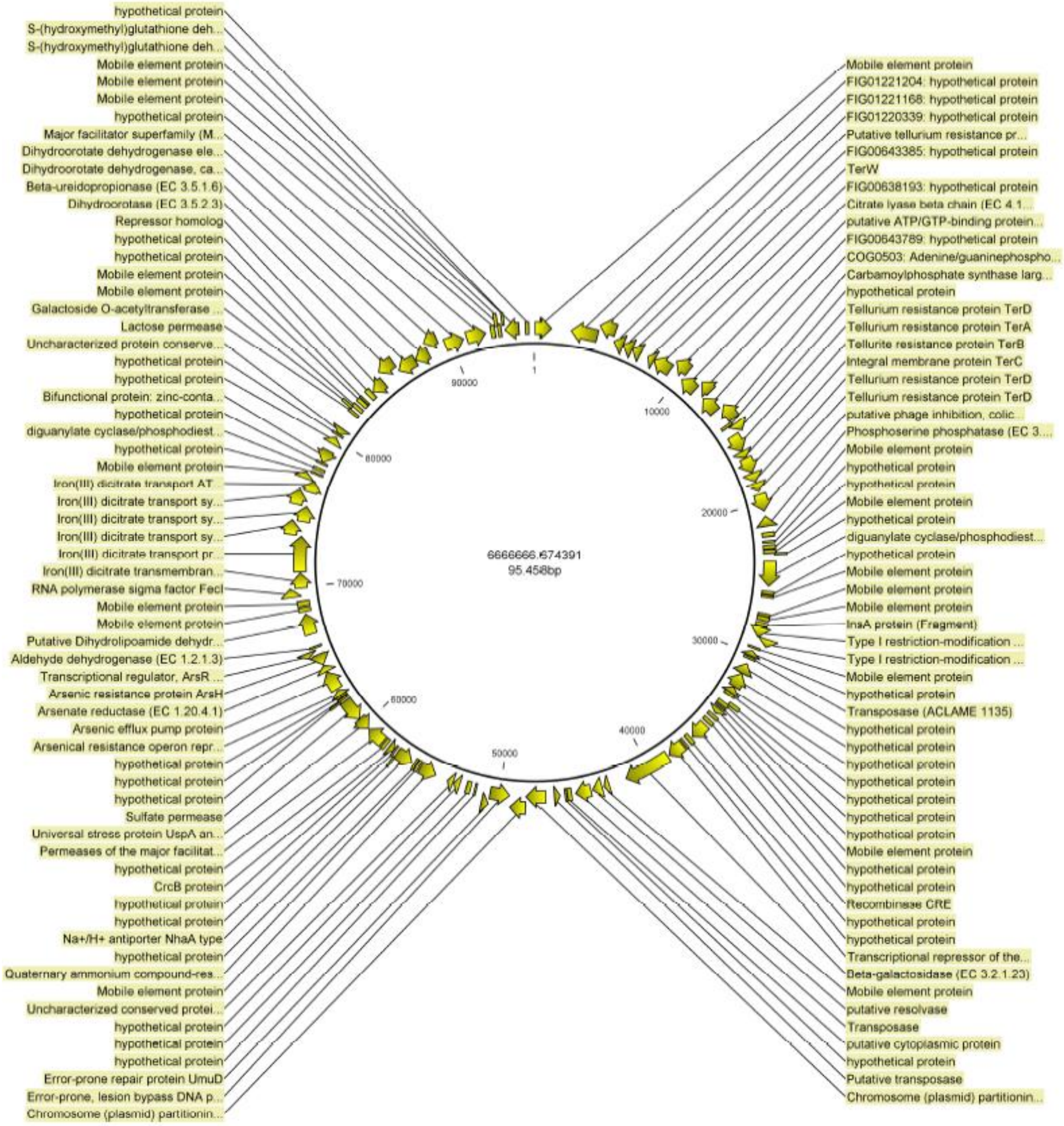

S.4.3. Plasmid present in the DO7785 *E. coli* isolate

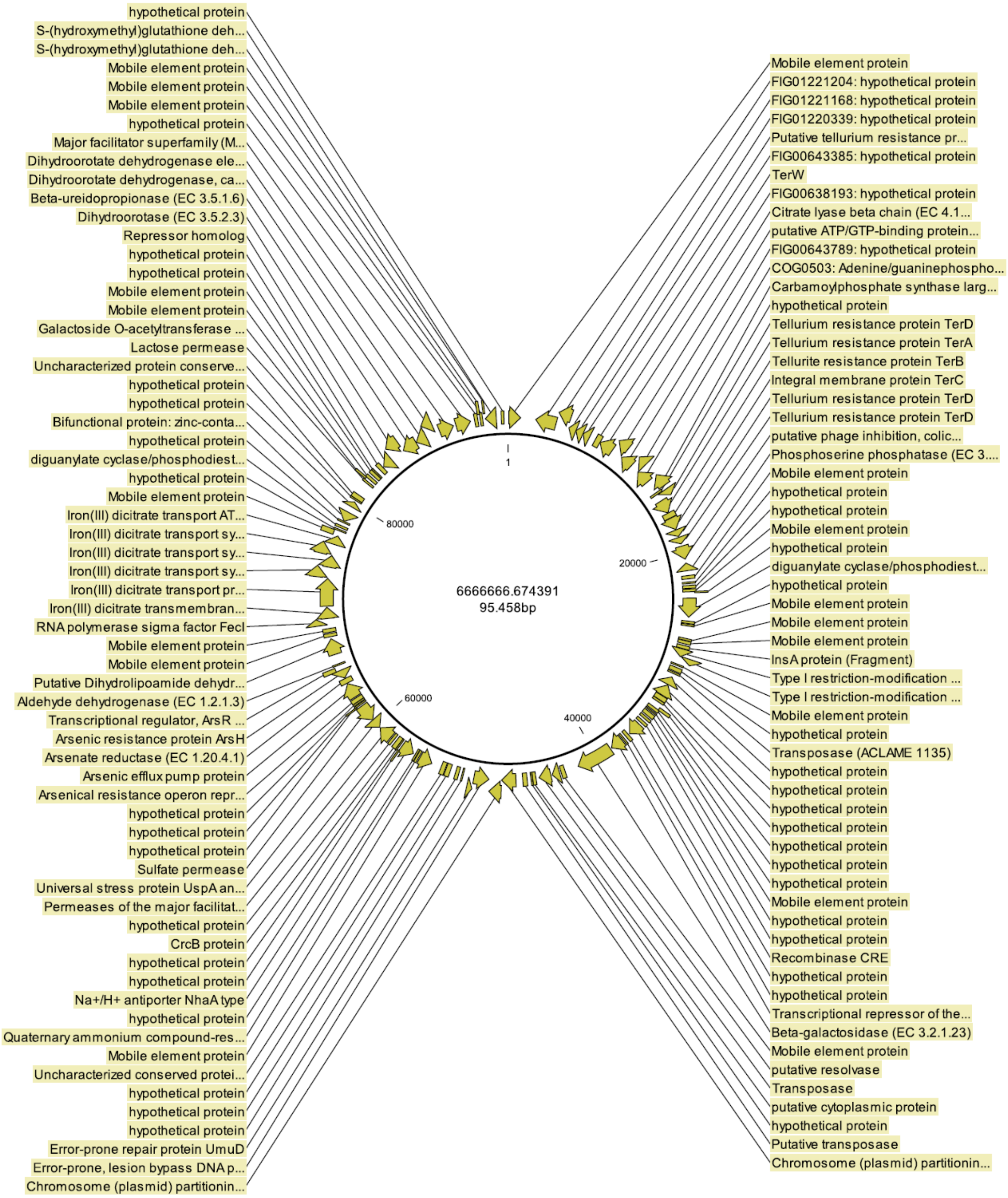

S.5. Virulence genes identified.

	ybtS	*mrkD*	*rmpA*	*iutA*	*allS*	*fur*	*fimF*	*ureA*	*uge*	*pgaC*	*wwzi*	*fimC*	*OmpA*	*IroN*	*WabG*	*hgpA*	*irp2*
**kp1**		■						■	■	■	■	■	■		■	■	
**kp2**		■						■	■	■	■	■	■		■	■	
**Kp3**		■						■	■	■	■	■	■		■	■	
**Kp4**		■						■	■	■	■	■	■		■	■	
**DO7785**				■	■		■						■				■
**1076**		■						■	■	■	■	■	■		■	■	
**2801**	■	■						■	■	■	■		■		■	■	■
**648**		■						■	■	■	■	■	■		■	■	
**5459**		■						■	■	■	■	■	■		■	■	

Identified virulence genes. The red squares indicate the presence of the gene while the squares indicate the absence of the gene; in order KP1-KP4 *K. pneumoniae* isolates from blood of patients with endocarditis, DO7785 *E. coli* isolate from patient with endocarditis and finally 1076, 2801, 648 and 5459 *K. pneumoniae* isolates from urine samples of patients with urinary tract infections. *ybtS* = Yersiniabactin; *mrkD* = Type 3 fimbria adhesin subunit MrKD; *iutA* = Aerobactin receptor; *allS* = Gene associated with allantoin metabolism; *fimH* = Fimbrial adhesin H; *ureA* = Urease subunit alpha; *uge* = UDP galacturonate – 4 – epimerase; *pgaC* = polysaccharide adhesin; *wzi* = Code for outer membrane protein WZI; *fimC* = Fimbrial adhesin C; *OmpA* = Outer membrane porine A; *wabG* = Lipopolysaccharide biosynthesis; *hgpA* = Hemoglobin and hemoglobin-haptoglobin binding protein; *irp2* = Iron regulatory protein.

S.6. Adherence of *Escherichia coli* and *Klebsiella pneumoniae* strains to cells lineage.

S.6.1. Adherence to Vero cell line

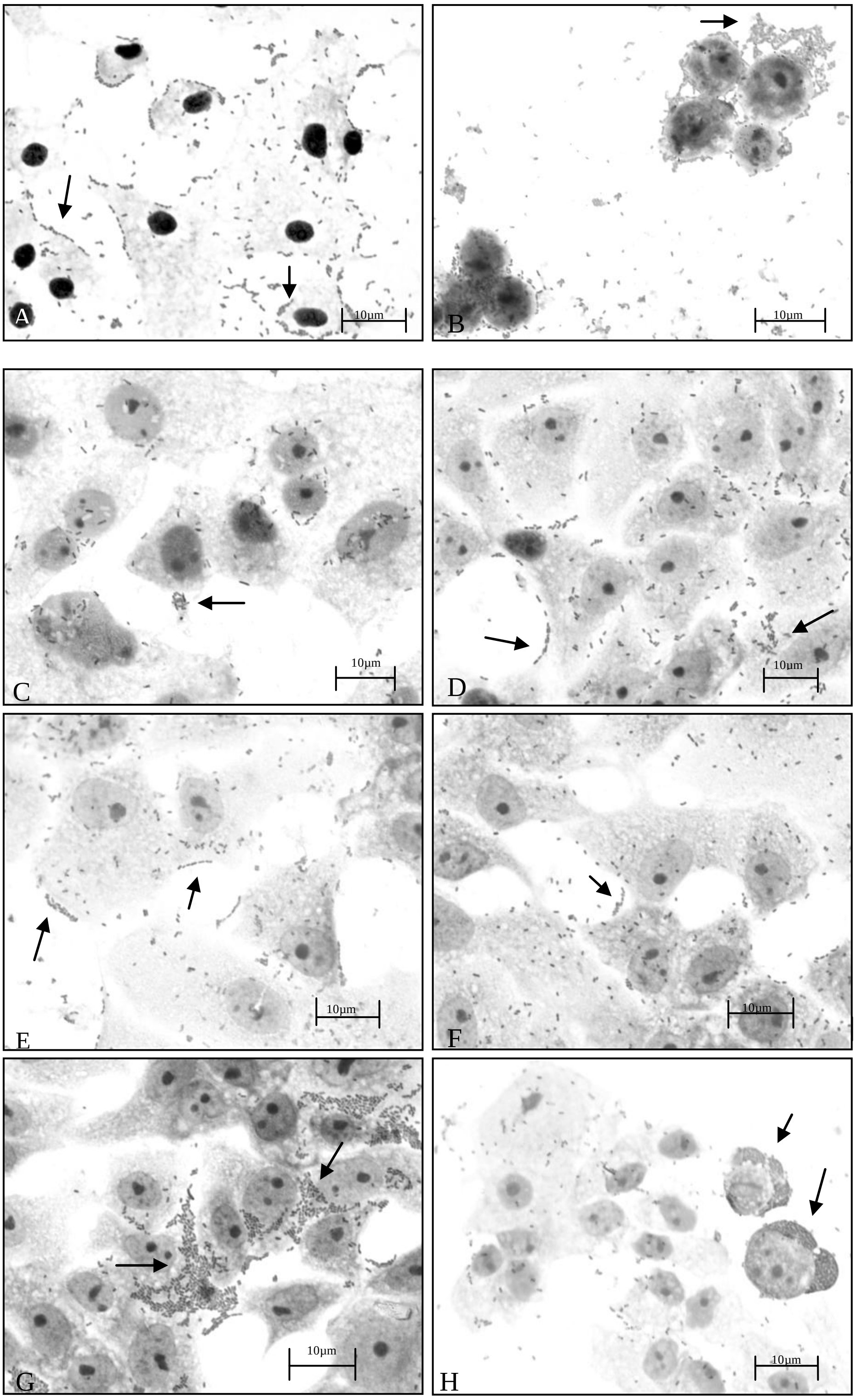

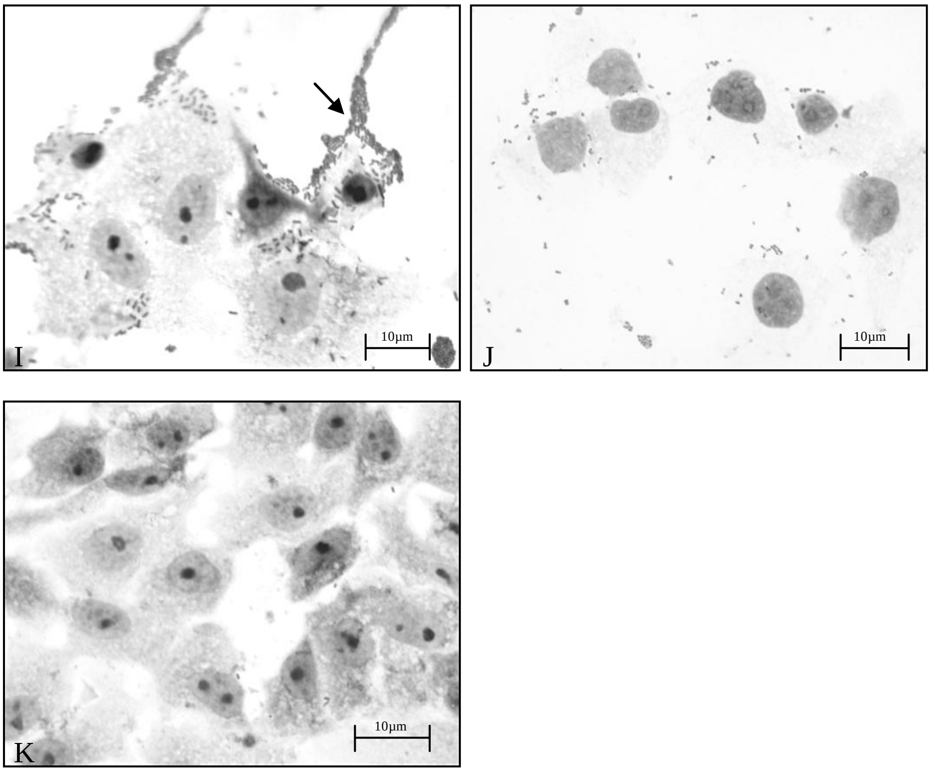

Adherence in Vero cell line. Figure a and b: The EAEC and UPEC strains, respectively, were used as positive control for adherence to Vero cell line (arrow). Figure c: The *E. coli* strain DO7785 showed adherence to Vero cell line when compared with control strains 042 and I64 (arrow). Figures from d to g: KP1, KP2, KP3 and KP4 isolates showed adherence to the lineage as in the strains respectively (arrow). Figures h and i: *K. pneumoniae* isolates from urine, 2801 and 1076 were able to adhere to Vero respectively (arrow). Figure j: The 5459 isolate showed poor adherence (arrow). Figure k: The 648 strain was not able to adhere to the lineage. a:042 (EAEC), b:I64 (UPEC), c:DO7785; d:KP1, e: KP2, f: KP3, g: KP4, h: 2801, i: 1076, j: 5459 and k: 648. Scale: 10 µm.

S.6.2. Adherence of *Escherichia coli* and *Klebsiella pneumoniae* strains in HEp-2 cell line

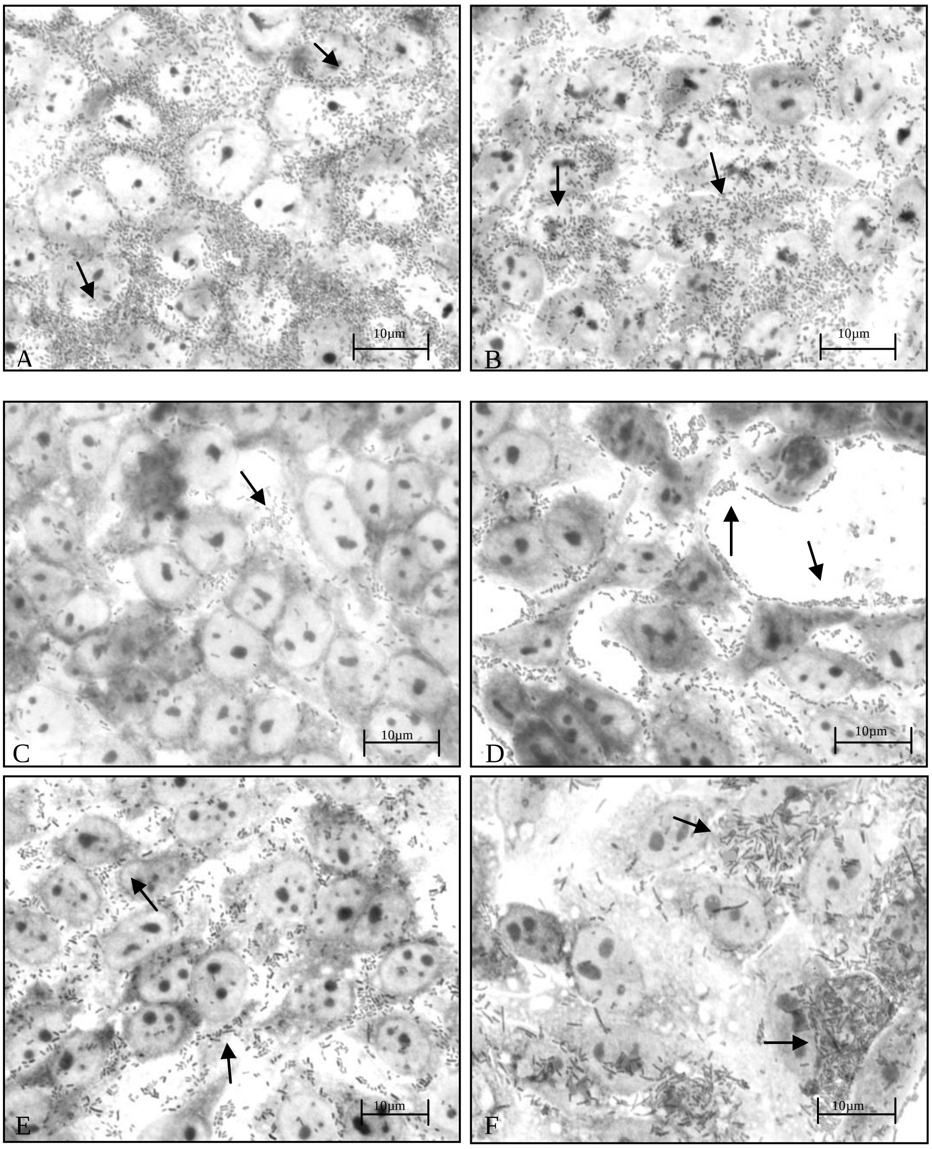

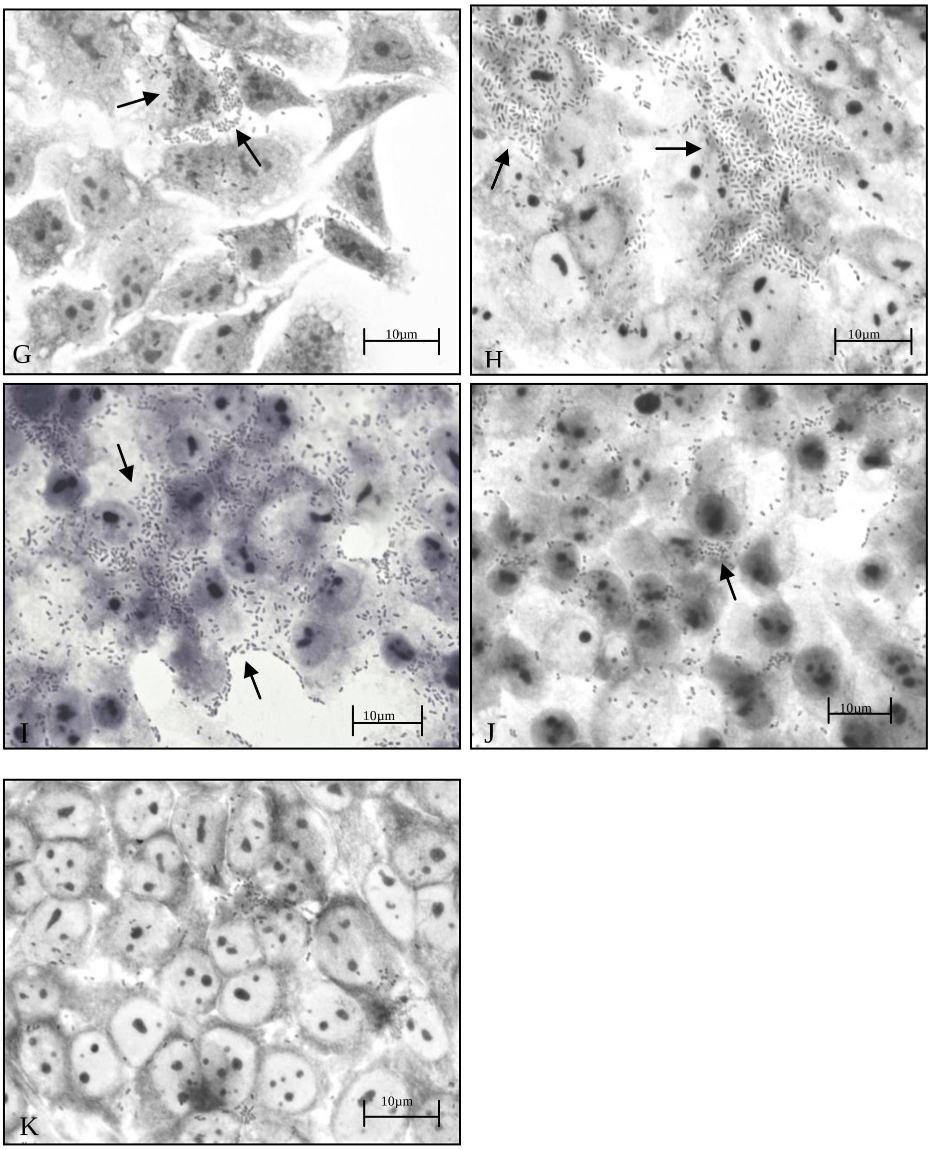

Adherence in HEp-2 cell line. Figure a and b: The EAEC and UPEC strains, respectively, were used as positive control for adherence to Hep-2 cell line (arrow). Figure c: The *E. coli* strain DO7785 showed adherence to Hep-2 cell line when compared with control strains 042 and I64 (arrow). Figures from d to g: KP1, KP2, KP3 and KP4 isolates showed adherence to the lineage as in the strains respectively (arrow). Figures h and i: *K. pneumoniae* isolates from urine, 2801 and 1076 were able to adhere to Hep-2 respectively (arrow). Figure j: The 5459 isolate showed poor adherence (arrow). Figure k: The 648 strain was not able to adhere to the lineage. a: 042 (EAEC), b:I64 (UPEC), c:DO7785; d:KP1, e: KP2, f: KP3, g: KP4, h: 2801, i: 1076, j: 5459 and k: 648. Scale: 10 µm.
